# The Response of the Root Apex in Plant Adaptation to Iron Heterogeneity in Soil

**DOI:** 10.3389/fpls.2016.00344

**Published:** 2016-03-21

**Authors:** Guangjie Li, Herbert J. Kronzucker, Weiming Shi

**Affiliations:** ^1^State Key Laboratory of Soil and Sustainable Agriculture, Institute of Soil Science, Chinese Academy of SciencesNanjing, China; ^2^Department of Biological Sciences, University of Toronto, TorontoON, Canada

**Keywords:** iron, root apex, morphology, physiology, plant

## Abstract

Iron (Fe) is an essential micronutrient for plant growth and development, and is frequently limiting. By contrast, over-accumulation of Fe in plant tissues leads to toxicity. In soils, the distribution of Fe is highly heterogeneous. To cope with this heterogeneity, plant roots engage an array of adaptive responses to adjust their morphology and physiology. In this article, we review root morphological and physiological changes in response to low- and high-Fe conditions and highlight differences between these responses. We especially focus on the role of the root apex in dealing with the stresses resulting from Fe shortage and excess.

## Introduction

Iron (Fe) is an essential element, critical to the key primary processes of photosynthesis, respiration, and nitrogen metabolism, and species differ greatly in how much Fe they require for optimal growth. Fe exists in two oxidation states, Fe^3+^ (ferric) and Fe^2+^ (ferrous), and rates of absorption and toxicities for the two forms differ significantly, typically with Fe^2+^ showing higher absorption and toxicity (Becana et al., 1998; Fageria et al., 2008; Brumbarova et al., 2015; Li et al., 2015b). Fe is present in excess in soils of low pH and oxygen tension (Connolly and Guerinot, 2002; Becker and Asch, 2005), while it is frequently limiting in alkaline and aerobic soils (Imsande, 1998; Eroglu et al., 2016). Therefore, knowledge of the mechanisms by which plants maintain Fe homeostasis is of great biological importance and agronomic significance. Root adaptations to Fe limitation and toxicity occur at both morphological and physiological levels. The root apex, the initiator organ of root tropisms, cell polarity, and fate (Baluška et al., 1990), represents the most probable site for sensing Fe limitation and excess. Here, we outline recent progress in our understanding of how plant roots respond to low and high Fe, and especially highlight the mechanisms involving the root apex in these responses.

## Morphological Changes

Plants can respond to suboptimal environmental conditions by growth redistribution within their root system architecture (RSA), a process referred to as the stress-induced morphogenic response (Potters et al., 2009; Zolla et al., 2010; De Smet et al., 2015). Nutrient availability can exert a profound impact on RSA by altering the number, length, angle, and diameter of roots and root hairs (for review, see Forde and Lorenzo, 2001; López-Bucio et al., 2003; Osmont et al., 2007; Giehl et al., 2014). By allocating carbon flow to facilitate directional root growth to more favorable soil patches, plants can respond to the heterogeneous availability of nutrients flexibly and rapidly (Zhang and Forde, 1998; Hermans et al., 2006; Giehl et al., 2012a,b; Gruber et al., 2013). Excess Fe has been shown to arrest primary root (PR) growth by decreasing both cell elongation and division, and to inhibit lateral root (LR) initiation in newly grown roots elongating during exposure to excess Fe, sparing proximal roots formed prior to excess-Fe exposure (Yoshida, 1981; Yamauchi and Peng, 1995; Li et al., 2012, 2015a,b; Reyt et al., 2015), resulting from direct contact of the root tip with Fe. Fe^2+^, the most toxic form, is increasingly present in lower soil strata, where low pH and anoxia prevail (Ratering and Schnell, 2000). Thus, an adjustment of RSA to restrict excessive Fe absorption in lower strata can prevent more serious Fe toxicity, while still allowing for the acquisition of essential nutrients in other parts of the root system. Supportive of this, excess Fe was seen to have no significant effect on LR formation in the proximal roots, with relatively stable LR number and length in this portion of the root system, to which the absorption of other nutrients can be delegated when other components of the root system are under stress, allowing controlled acclimation to nutritional stress (Heil and Baldwin, 2002). Furthermore, root gravitropism can rapidly alter root growth orientation when the root tip is exposed to Fe stress, and this can modify the direction of root growth away from the stress stimulus (Wisniewska et al., 2006; Zou et al., 2012, 2013; Li et al., 2015a). By contrast, to cope with Fe deficiency, plants can increase the exchange surface of the root system, thus enhancing its foraging capacity. A shortage in Fe triggers the formation of ectopic root hairs at positions normally occupied by non-hair cells and leads to bi-furcated root hairs with two tips (Schmidt et al., 2000). This peculiar phenotype plays an important role in Fe uptake by increasing the absorptive surface layer of the root. Similarly, Gruber et al. (2013) suggested that the length of the PR and the number of LRs both increase under moderate Fe deficiency, while they drastically decrease under severe Fe deficiency. Furthermore, Giehl et al. (2012a,b) showed Fe limitation affects RSA by regulating root-tip auxin and Fe content to promote LR elongation in Fe-enriched zones. Thus, at least under moderate Fe deficiency, the plasticity in the root system’s response offers the adaptive advantage of exploiting more distantly located Fe patches in soils; however, under severe Fe deficiency, acclimation proceeds via a growth-dependent pathway that transiently arrests root elongation and number to reduce nutrient demand (Seguela et al., 2008). This is similar to what is observed under phosphate (Pi) deficiency: Pi deprivation also requires continued root growth, whereas an inhibition of cell-cycle activity represses Pi uptake in response to decreased Pi demand (Lai et al., 2007).

## Physiological Changes

**FIGURE 1:**
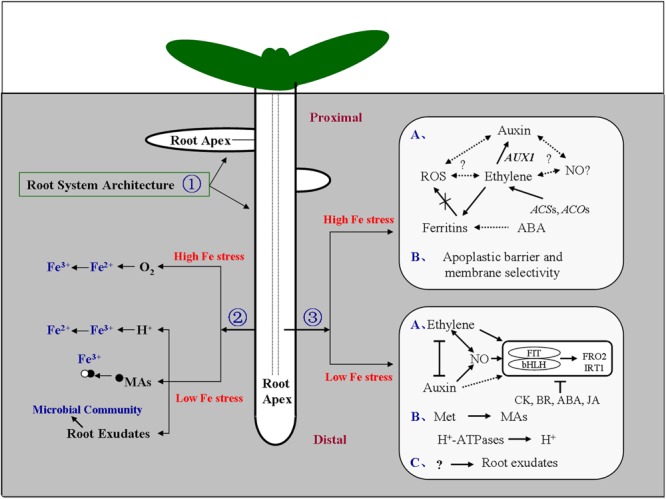
**Model of the signaling responses under iron (Fe) stress in the root.** Signal locations and physiological pathways of Fe stress are shown. The soil distribution of Fe is highly heterogeneous, Fe stress occurs in both low-Fe and high-Fe environments. To cope with this heterogeneity, plant roots, and in particular the root apex, engage an array of adaptive responses to adjust their morphology and physiology. ① Dynamic changes in root system architecture (RSA) could regulate the exchange surface of the root to enhance or restrict Fe absorption in low-Fe and high-Fe environments, respectively, or alter root growth orientation. ② Roots could secrete protons, and acids such as mugineic acids, and regulate the microbial communities in the rhizosphere to enhance Fe available under deficiency, and facilitate diffusion of molecular oxygen to the root medium to oxidize Fe^2+^ to Fe^3+^ under Fe^2+^ excess. ③ Hormones and other molecules could act in concert, and either downstream or upstream of one other, to affect the Fe-uptake gene expression, tissue Fe homeostasis, and cell-wall components, in response to low-Fe or high-Fe environments (Brumbarova et al., 2015; Li et al., 2015a,b). The root apex represents the most probable site for sensing Fe limitation and excess, and plays an important role in perceiving and transducing Fe signals into physiological and developmental responses. Arrows indicate promotion, and perpendicular lines indicate inhibition. Broken arrows indicate a potential effect or interaction. (A–C) represent each response pathway. ABA, abscisic acid; ACO, 1-aminocyclopropane-1-carboxylic acid oxidase; ACS, 1-aminocyclopropane-1-carboxylic acid synthase; AUX1, auxin-resistant 1; bHLH, basic helix-loop-helix; BR, brassinosteroids; CK, cytokinins; FIT, Fer-like Fe-deficiency-induced transcription factor; FRO, ferric chelate reductase; IRT, Fe-regulated transporter; JA, jasmonic acid; MAs, mugineic acid family; Met, methionine; NO, nitric oxide; O_2_, molecular oxygen; ROS, reactive oxygen species. Proximal, the root portion that existed at the time of transfer; Distal, the root portion that was established after transfer.

To ensure effective Fe acquisition from the rhizosphere under Fe deficiency, plants have developed two principal strategies: Strategy I and Strategy II (Marschner et al., 1986; Romheld and Marschner, 1986; Schmidt, 2003). The initial step in strategy I is to acidify the rhizosphere, through increased proton eﬄux via plasma-membrane (PM) H^+^-ATPases (Schmidt, 2006; Santi and Schmidt, 2009). The root tip is an important regulatory region modulating H^+^ secretion (Haruta and Sussman, 2012; Xu et al., 2012). In *Arabidopsis, FRO2* (ferric chelate reductase, FRO) and *IRT1* (Fe-regulated transporter, IRT) genes encode a ferric reductase and an Fe transporter engaged in Fe acquisition from acidified media, respectively (Eide et al., 1996; Robinson et al., 1999; Jeong and Guerinot, 2009). An Fe-regulated (Fer)-like Fe-deficiency-induced transcription factor (FIT), heterodimerizes with at least four *bHLH* transcription factors, and directly binds to a subset of Fe-regulated genes in the root, including *FRO2* and *IRT1*, driving their up-regulation under low Fe (Colangelo and Guerinot, 2004; Walker and Connolly, 2008; Sivitz et al., 2012; Wang et al., 2013). The interplay between hormones [ethylene, auxin, jasmonic acid (JA), cytokinins (CK), brassinosteroids (BR), abscisic acid (ABA)] and nitric oxide (NO) is also critical in Fe-deficiency signaling (see the reviews by Jeong and Guerinot, 2009; Brumbarova et al., 2015; **Figure 1**). In grasses, a different strategy for Fe uptake has developed, known as Strategy II (Takagi et al., 1984; Marschner et al., 1986; Shi et al., 1988; Zhang et al., 1989; Kobayashi and Nishizawa, 2012). Strategy-II plants synthesize and release compounds such as mugineic acids (MAs) into the rhizosphere to solubilize Fe and chelate ferric Fe (Shi and Liu, 1991; Walker and Connolly, 2008; Kobayashi and Nishizawa, 2012); *S*-adenosylmethionine is the first substrate in the mugineic acid biosynthesis pathway (Ma et al., 1995; Mori, 1999). Exudation of organic acids from the growing root tip is well documented in other stress-resistance mechanisms, such as Al stress (Kochian et al., 2004; Sivaguru et al., 2013). Recently, several studies have shown that soil microbial activity, influenced by root exudates, can further impact Fe acquisition (Rroco et al., 2003; Jin et al., 2010). Phenolics have also been identified in Fe-deficiency-induced root exudates (Jin et al., 2014), although the precise constituents remain poorly understood. Additional complexities arise when plants are mycorrhizal; in maize, for instance, strong induction of two Fe transporters, *OPT8a* and *OPT8b*, bypassing Strategy II, by mycorrhizal colonization was documented (Kobae et al., 2014). Thus, much needs to be learned about rhizospheric microbial partners and their roles in Fe acquisition.

Studies in rice growing under flooded conditions suggest a development of oxidic mechanisms to cope with adverse Fe-toxic conditions. Rice roots diffuse molecular oxygen to the root medium, rendering the rhizosphere significantly more oxidative than bulk soil (Chen et al., 1980a,b). Rhizospheric oxidation to Fe^3+^ ensues, which helps keep root-medium Fe^2+^ low and is instrumental to the formation of an oxidized ‘iron plaque’ layer on the root surface to limit Fe uptake. Furthermore, limiting excessive tissue Fe accumulation under excess Fe supply engages apoplastic and symplastic mechanisms. About 90% of Fe^2+^ is typically intercepted by the root apoplast, although the mechanism of interception is only partially understood (McLaughlin et al., 1985). One explanation is that Fe^2+^ availability in the apoplast is reduced via alkalinization of apoplastic pH (Kosegarten et al., 2004), which affects both Fe^2+^ mobility and chemical stability, and this alkalinization can be affected by ethylene (Staal et al., 2011). Another possible hypothesis relates to the cation-exchange capacity of the cell wall. The cell wall acts as a major pool of calcium, aluminum, cadmium, and other cations, cell-wall components with negative surface charges (pectin and hemicelluloses) possess cation-adsorption capacities, and cations compete for these surface charges with variable potency (Kronzucker et al., 1995). It has been reported that ethylene, auxin, and NO play roles in the cell-wall adjustment to stress (Xiong et al., 2009; Tsang et al., 2011; Zhu et al., 2013). Fe^2+^ can also be excluded at root-cell membranes (Tadano, 1975). Additionally, plants can sequester Fe^2+^ in root-cell vacuoles and in the multimeric protein ferritin (Connolly and Guerinot, 2002; Majerus et al., 2007, 2009). In maize (*Zea mays* L.) and *Arabidopsis*, regulation of ferritin gene expression in response to Fe excess occurs at the transcriptional level (Briat et al., 2010; Li et al., 2015b), and involves regulatory pathways mediated by ABA, reactive oxygen species (ROS), and ethylene. However, Majerus et al. (2009) showed that a signaling pathway leading to the induction of ferritin synthesis depended neither on ABA nor oxidative stress in African rice.

## Response Mechanism of the Root Apex

Identifying the stress-sensing site is as critical under Fe stress as under other stresses. As the root apex is the first part of the plant to come into contact with previously unexplored soil regions, the tip represents the most probable candidate (Dat et al., 2004; Baluška et al., 2010). Growth-inhibitory effects of low phosphate (Svistoonoff et al., 2007; Ward et al., 2008) or elevated aluminum (Ryan et al., 1993; Jones and Kochian, 1995; Baluška and Mancuso, 2013; Sivaguru et al., 2013) are sensed by the root tip. The root tip transition zone was also suggested as a primary site in sensing and transducing nitrate signals (Trevisan et al., 2015). Some receptors for environmental signals have been reported to be strongly expressed in the root tip, such as, *LPR1*, which senses low Pi (Svistoonoff et al., 2007; Müller et al., 2015). The root tip has received much attention devoted to the action of hormones and other molecules. Plant hormones and other molecules could act in concert, and either downstream or upstream of one other. With more evidence accumulating supporting the involvement of different molecules in Fe signaling, a necessary task will be to determine how they integrate into the larger signaling network. This will also contribute to our understanding of exactly how the root tip can act as a signaling-response nexus. Fe excess leads to a significant decrease in PR length, and recent results have shown that contact of the PR tip with Fe is both necessary and sufficient for PR inhibition (Zhang et al., 2011, 2012; Li et al., 2015b). Fe excess could modulate the H_2_O_2_/O_2_^⋅-^ balance, decreasing O_2_^⋅-^ in the root tip proliferation zone and increasing H_2_O_2_ production in the transition zone, to arrest PR growth (Reyt et al., 2015), as suggested by the model by Tsukagoshi et al. (2010), which correlates PR growth with the relative distribution of O_2_^⋅-^ and H_2_O_2_ in the tip. Meanwhile, ethylene evolution is enhanced by upregulating expression of *ACS* and *ACO* genes in the root tip and protects root growth under Fe toxicity by regulating tissue Fe homeostasis (Harahap et al., 2014; Li et al., 2015b). Despite the well-known functions of ethylene and ROS signaling during a variety of abiotic stresses, whether ethylene acts alone or in conjunction with ROS in root-tip acclimation to Fe excess remains to be elucidated. It has been reported that enhanced NO generation in the root transition zone is required for maintaining root growth under cadmium stress (Alemayehu et al., 2015). However, a clear role for root-tip NO in regulating root growth under Fe excess has not yet bee established (see below). The root tip is also the primary sensing site for the LR formation response to excess Fe, and reduced LR formation in response to excess Fe was found to be partially related to auxin levels, while root-tip *PIN2* protein expression and ethylene-related *AUX1* functions were shown to play a positive role on LR formation under Fe excess (Li et al., 2015a). Furthermore, LR development also requires ROS signaling (Manzano et al., 2014). *Arabidopsis* seedlings exposed to oxidative stress-inducing agents display clear modifications in auxin homeostasis, suggesting a possible crosstalk between ROS and auxin signals (Cheng et al., 2011; Yuan et al., 2013). This may constitute a signaling intersection point within the root tip to meditate intelligent growth responses to Fe excess. The *PIN2* gene, critical to routing signals to either the root or shoot apex, is seen as a general stress target due to its strong sensitivity to a variety of environmental stresses, such as cold, salt, and aluminum, supporting root stress avoidance (Baluška et al., 2010). Auxin distribution within the root under Fe stress responds to alterations in *PIN2* gene expression in the root tip (Li et al., 2015a), and this may modify the direction of root growth away from the stress stimulus (Sun et al., 2008). Furthermore, ROS and NO signaling in the root apex are also implicated as an early response in gravitropic acclimation (Mugnai et al., 2014). Fe^2+^ presence is increased by hypoxic or anoxic conditions (Mongon et al., 2014), and the transition zone seems to be the most sensitive region of the root to oxygen deprivation (McLamore et al., 2010; Larsen et al., 2015). Moreover, local NO peaks in the transition zone are essential to the successful acclimation of the entire root to oxygen deprivation (Mugnai et al., 2012)

Similarly, under Fe deficiency, Giehl et al. (2012a,b) found that locally supplied Fe evokes RSA modifications and affects the local symplastic Fe gradient in LRs, upregulating the *AUX1* gene to accumulate auxin in LR tips as a prerequisite for LR elongation. Thus, *AUX1* may represent a check-point at which systemic and local nutritional signals are integrated into the overall root developmental program (Giehl et al., 2014). Proton secretion is regulated in the response to Fe deficiency and maintains or promotes PR elongation and root hair development (Santi and Schmidt, 2009; Yang et al., 2010; Xu et al., 2013). Several studies have shown that the root tip plays an important role in the response to Fe stress by mediating proton and organic acids secretion ((López-Millán et al., 2000; Abadia et al., 2002). In addition, NO levels are altered in response to Fe availability in the root tip and have been invoked in signal transmission (Chen et al., 2010; Romera et al., 2011). Although NO interacts with ethylene and auxin (Freschi, 2013), the details of this mechanism remain largely unknown. Reporter gene studies have shown that ethylene and auxin can act antagonistically in regulating the topology of *IRT1* gene expression along the root (Blum et al., 2014). These suggest that the root apex serves as the sensing site initiating the growth response to both low and excess Fe, shortly after the root apex reaches the heterogeneous Fe zone, triggering a sequence of signaling events. To further understand the interactions of the various signaling pathways and of the gene-regulatory network in the root-tip response to Fe, future work will need to combine genomic and genetic approaches. For example, Trevisan et al. (2015) confirmed the root tip transition zone as a critical zone in sensing nitrate using genome-wide studies and postulated the contribution of NO to the nitrate-induced transcriptional response in the transition zone. Moreover, Satbhai and Busch (2014) used a set of 450 natural accessions of *Arabidopsis* to identify genes that quantitatively regulate root growth responses to Fe deficiency using genome-wide association mapping, and more than 20 genomic loci were found to be significantly associated with changes in root growth rate upon Fe deficiency.

## Conclusion

Recently, important progress has been made in our understanding of how plants maintain Fe homeostasis in response to heterogeneous Fe supply in soils. Many of the morphological and physiological responses are now understood, and various regulatory factors have been shown to take part in the sensing of soil-Fe stress, both in low-Fe and in high-Fe environments, and many of the components of the signaling pathways engaged have been mapped out (**Figure 1**). This working model should provide important new insight into plant responses to heterogeneous Fe supply. Our knowledge of the molecular components involved in the Fe-stress response is in its infancy, and details of signal transduction, such as the precise identification of sensors and transcription factors, remain a challenge. The root apex has emerged as the primary sensing site for Fe stress, and it is hoped that future work will elucidate how apex sensing integrates into whole-plant signaling and translates into an intelligent root-system-architecture response to Fe stress.

## Author Contributions

GL drafted the manuscript. GL, HK, and WS revised the manuscript.

## Conflict of Interest Statement

The authors declare that the research was conducted in the absence of any commercial or financial relationships that could be construed as a potential conflict of interest.
